# The Stream Device—A Retrospective Review of 51 Cases

**DOI:** 10.3390/jcm12196384

**Published:** 2023-10-06

**Authors:** Karlis Kupcs, Patricija Sproge, Katrina Kupca, Pervinder Bhogal

**Affiliations:** 1 Department of Neuroradiology, Pauls Stradins Clinical University Hospital, LV-1002 Riga, Latvia; kkupcs@gmail.com (K.K.); patricija.sproge@gmail.com (P.S.); katrina.kupca@gmail.com (K.K.); 2 Department of Radiology, Riga Stradins University, LV-1007 Riga, Latvia; 3 Department of Interventional Neuroradiology, The Royal London Hospital, Barts NHS Trust, London E1 1BB, UK

**Keywords:** stream device, mechanical thrombectomy, ischaemic stroke

## Abstract

Mechanical thrombectomy is the gold-standard treatment for patients that have suffered large-vessel occlusion (LVO) stroke. Various different stent-retrievers, aspiration catheters, and techniques have been developed to perform this procedure. We present our initial results regarding the Stream device. Materials and Methods: We performed a retrospective review of a prospectively maintained database at our high-volume centre to identify all patients treated with the Stream device between February 2021 and January 2023. We recorded baseline demographics, NIHSS, ASPECT scores, eTICI scores, complications, and 90-day mRS. Results: We identified 51 patients, 49.0% of whom were male (*n* = 25), with a median age of 73 (range: 51–89) and a median NIHSS score of 17 (range 4–22), and 68.6% received IV tPA. The median ASPECT score was 10 (range 6–10). Hyperdense clots were seen in 34 cases (66.7%), with a mean clot length of 12 ± 6.2 mm (range 2–26 mm). Clots were located in the anterior circulation in 49 patients. The standard Stream device was used in 78.4% of cases, with Stream 17 being used in 19.6% of cases. The FPE was observed in 25.5% of cases (*n* = 13), with the mFPE being seen in 31.4% of cases (*n* = 16). A final eTICI score of ≥2b was achieved in 90.2% of cases (*n* = 46), and eTICI 2c/3 was seen in 84.3% of cases (*n* = 43). Furthermore, 24 h CT scans showed that the median ASPECT score was 8 (range 0–10). Good functional outcomes at 90 days (mRS ≤ 2) were achieved in 21.6% of cases (*n* = 11). Conclusions: The Stream device shows acceptable rates of FPE and mFPE compared to existing devices. Further larger studies are required alongside an understanding of the optimal technique for this device’s use.

## 1. Introduction

Mechanical thrombectomy (MT) has become the gold-standard treatment for acute ischaemic stroke (AIS) caused by large-vessel occlusion (LVO), with studies further demonstrating the efficacy of this procedure in treating patients presenting beyond 6 h [1,2], in relation to posterior circulation, and, more recently, in treating patients with large-core strokes (with ASPECT scores of 3–5) [3,4,5]. A variety of studies assessing the impact of MT in more distal locations are currently underway, with the expectation that the indications for MT will continue to expand.

Recent advances in our understanding related to clot pathophysiology and structure, clot/stent interactions, and clot/vessel wall interactions have improved our knowledge base regarding ischaemic stroke [6,7,8,9,10]. Over the last decade, there has been a significant improvement in our understanding of the various clot types [6], the interaction between stent-retrievers and clots [7,8,9], and how both stent-retrievers and clots interact with the vessel wall and in combination during retrieval [10]. This improvement in our understanding has led to the development of stent-retrievers with novel designs [11,12,13,14,15], with the synchronous development of novel aspiration catheters [16,17,18] and various techniques for optimising the MT procedure and first-pass recanalisation.

Manually controlled, expandable, braided devices have been clinically used for the treatment of aneurysms [19,20,21,22,23] and, more recently, cerebral vasospasm [24,25]; however, their development and use in AIS are more recent occurrences. The Stream (Perflow Medical, Tel Aviv, Israel) device is a novel device with a braided structure that, via an actuator at the handle, can be expanded or contracted by the operator.

Currently, there are no publications that have examined the safety and efficacy of this novel device. In this article, we present our initial experience of the use of the Stream device to treat patients with AIS.

## 2. Materials and Methods

### 2.1. The Stream Device

**Figure 1:**
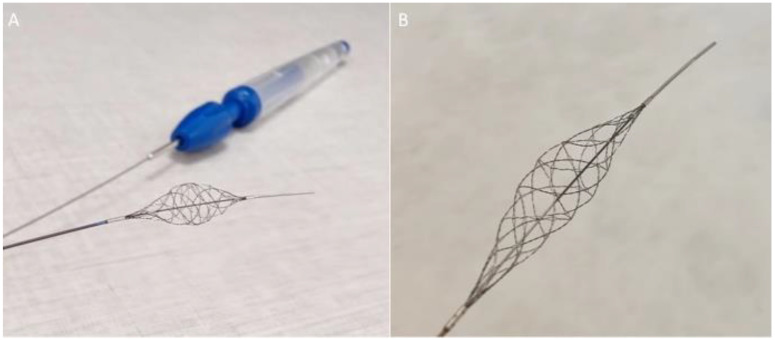
The Stream device consists of an actuator handle that allows for the precise control of the Cerebral Net ((**A**) and magnified image in (**B**)). At the distal end of the device, there is an atraumatic, radiopaque wire tip (**B**).

The Stream device is a dynamic neuro-thrombectomy braided net, or an adjustable stent retriever, which is controlled by a proximal handle (Figure 1). At the distal end of the device, there is a 10–13 mm (depending on the device model) atraumatic, radiopaque wire tip. There are 6–8 (depending on the device model) paired wires that form the expandable Cerebral Net™, and these can be expanded from 0.4 to 6 mm depending on the model of device chosen. The actuator control handle has two operation modes: auto-lock mode for stepwise radial expansion and free mode for continuous adjustments that can also provide tactile feedback. In auto-lock mode, each ‘click’ of the handle results in an incremental controlled expansion of the device. There are currently three models:

- Stream;
- Stream XL;
- Stream 17.

Stream and Stream XL are compatible with 0.021-inch-inner-diameter microcatheters, whereas Stream 17 is designed for 0.017-inch-inner-diameter microcatheters. In terms of length, Stream has a maximum braid length of 33 mm, Stream XL has a maximum braid length of 38 mm, and Stream 17 has a maximum braid length of 25 mm. The overall effective length of the Stream is 193 cm.

### 2.2. Patient Selection

We performed a retrospective review of prospectively maintained databases at a high-volume centre to identify all patients treated with the Stream device between February 2021 and January 2023. The retrospective nature of this study precluded the requirement of ethical approval at the participating centre.

### 2.3. Study Population

The inclusion criteria included the following:

- Age ≥ 18;
- National Institutes of Health Stroke Scale (NIHSS) score of ≥4;
- ASPECT score ≥ 5;
- LVO on CT angiography;
- Pre-morbid mRS of 0–2;
- Life expectancy of >6 month.

The Stream device was the first device used for treatment of the intracranial occlusion.

Patients were excluded if another device or technique (aspiration) was used as the initial strategy for the MT procedure or if there was an incomplete dataset (aside from the 90-day mRS).

All patients underwent non-enhanced CT and CT Angiograms from the arch to the vertex prior to the MT procedure. Patients were eligible for MT if their ASPECT score was ≥5, their baseline mRS was ≤2, and their life expectancy was greater than 6 months (in the case of a known underlying malignancy), with no upper limit on age. All patients were given IV tPA if they met the criteria.

### 2.4. Endovascular Procedure

Patients were treated either with local anaesthesia or general anaesthesia as per local standard practice.

The exact equipment used varied between the different operators; however, a distal aspiration catheter, typically a 6 Fr Sofia (Microvention, Aliso Viejo, CA, USA), was used in all cases and was typically introduced via a standard guide catheter such as a NeuronMax (Penumbra, Alameda, CA, USA).

Successful reperfusion was defined as eTICI ≥ 2b (67%). In addition, first-pass effect (FPE) and modified FPE were defined as eTICI ≥ 2c and as eTICI ≥ 2b, respectively, after the first thrombectomy attempt.

### 2.5. Post-Procedure

Post procedure, using non-enhanced CT imaging was routinely performed at 24 ± 6 h unless there was a sudden deterioration in a patient’s consciousness level, prompting an earlier scan. In cases of intracranial or extracranial stenting, a CTA was also performed.

The 90-day mRS was recorded via telephone interview or clinic review conducted by a trained stroke physician.

The data recorded included the demographics (age, gender, underlying medical conditions, admission NIHSS score, etc.), use of IV tPA, radiological findings (including the ASPECT score prior to MT), clot location, endovascular procedural information, and relevant timing. The eTICI score was recorded after the first pass of the device and at the end of the procedure alongside any complications and the 90-day mRS where available.

## 3. Results

### 3.1. Baseline Demographics

In total, 51 patients met our inclusion and exclusion criteria. The median age of the participants was 73 (range 51–89), and 49.0% were male (*n* = 25). Pre-existing hypertension was common (*n* = 40, 78.4%), diabetes mellitus was less common (*n* = 13, 25.5%), and atrial fibrillation was noted in just over half of the patients (*n* = 26, 51%). All patients were recorded as having a baseline mRS of 0 prior to the MT procedure. The results are summarised in Table 1.

### 3.2. Clinical and Angiographic Results

The median NIHSS score at presentation was 17 (range 4–22), and 68.6% received IV tPA prior to MT. The most common suspected cause of a stroke was thought to be cardioembolic (52.9%). Right-sided LVOs were more frequently seen (56.9%), and eight cases involved tandem lesions (15.7%). The median ASPECT score upon conducting a plain CT scan was 10 (range 6–10). Hyperdense clots were seen in 34 cases (66.7%), with a mean clot length of 12 ± 6.2 mm (range 2–26 mm). The vast majority of clots were located in the anterior circulation, with only two posterior basilar occlusions (3.9%).

The results are summarized in Table 2.

### 3.3. Procedural Outcomes

**Figure 2:**
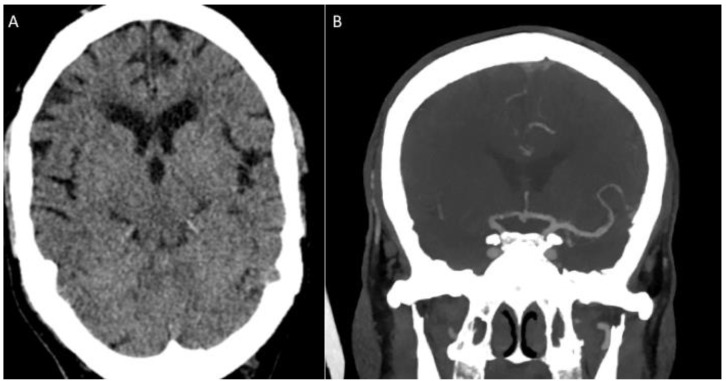
A male patient with 2 h history of acute left-sided weakness and an NIHSS score of 17. Non-contrast CT scan (**A**) revealed some subtle loss of grey–white matter differentiation involving the lentiform nucleus (ASPECT score 9), and CT angiography confirmed a right terminal ICA and M1 occlusion (**B**).

**Figure 3:**
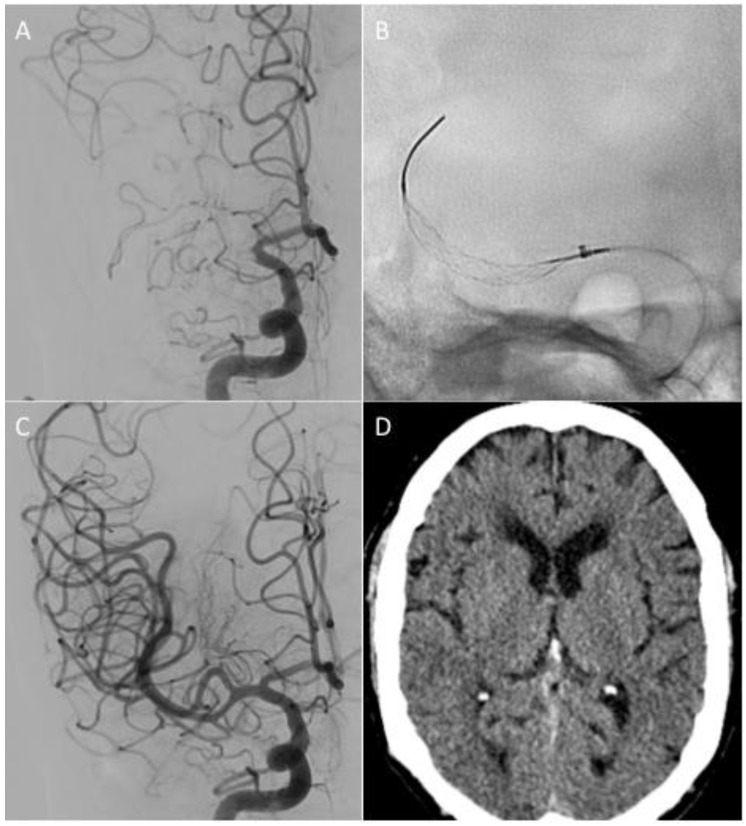
Angiography confirmed the terminal right ICA and M1 occlusion (**A**). The thrombus was crossed using a Headway 21 microcatheter, and a Stream device was expanded for 5 min (**B**). Withdrawal into the aspiration catheter was performed, during which the first-pass effect was observed (eTICI 2c) (**C**). Non-contrast CT scan of the head performed at 24 h demonstrated no evidence of haemorrhage and an ASPECT score of 8 (**D**).

The vast majority of patients were operated on under local anaesthesia (*n* = 49, 96.1%). The standard Stream device was used as the first-line device in the majority of procedures (*n* = 40, 78.4%), with the smaller Stream 17 used in 19.6% of cases (*n* = 10). The first-pass effect (FPE) was seen in 25.5% of cases (*n* = 13), with a modified FPE seen in 31.4% (*n* = 16) (Figure 2 and Figure 3). Distal embolisation was seen in 5.9% (*n* = 3) of cases, and an embolus in new territory was not seen in any cases.

In 19.6% (*n* = 10) of cases, Stream could not be used to remove the occlusion, and bailout with alternative devices/techniques was required. An intracranial stent was permanently implanted in one of these cases (1.9%). Carotid stents were implanted in two cases (3.9%).

The median number of passes when only Stream was used was 2 (range: 1-4), with a mean number of passes equal to 2.0 ± 0.92. At the end of the procedure, after all the devices and techniques had been used, a final eTICI score of ≥2b was achieved in 90.2% of cases (*n* = 46), with eTICI 2c/3 seen in 84.3% (*n* = 43) of cases. The results are summarised in Table 3.

### 3.4. Follow-up Imaging and Clinical Results

A follow-up CT scan performed at 24 h was available for all patients. The median ASPECT score was 8 (range 0–10). Subarachnoid haemorrhage was seen in eight patients (15.7%), and seven patients developed sICH (13.7%), three of whom also had SAH. The 90-day mRS was available for all patients, and a good functional outcome (mRS 0–2) was achieved in 21.6% of cases (*n* = 11), with an mRS of 6 in 43.1% of cases. The results are summarised in Table 4.

## 4. Discussion

In our initial experience with the Stream device, we observed a similar rate of efficacy with respect to FPE and mFPE when compared to other frequently used stent-retrievers such as the Solitaire (Medtronic) [26,27,28,29] and Trevo (Stryker) stent-retrievers [30,31,32], whose efficacy averages between 25 and 40%.

The Stream device has several potential technical advantages that come into effect during a mechanical thrombectomy. Unlike standard stent-retrievers, this device is under the direct control of the operating physician in the sense that it does not automatically expand like a standard device. Therefore, if the Stream device has been unsheathed in a sub-optimal position, it can be repositioned to optimise the clot/device interaction. Although one can achieve the same result with standard stent-retrievers, a potential drawback is that they will immediately expand upon unsheathing, and this will not only compress the clot but also result in microthrombi that have the potential to travel distally [10]. Similarly, the closed nature of the distal end of the Stream device’s braided net may also act as a distal clot catcher that prevents, at least to a degree, the loss of distal thrombi during the expansion of the device, as was recently demonstrated with another device [33]. Interestingly, in our study, distal emboli were only seen in 5.9% of cases despite having been reported in ≈25% of cases for devices with distal clot catchers [12]. It is also noteworthy that the average clot length in the three cases where distal clot embolisation occurred was 17.7 ± 6.8 mm, which was considerably longer than the average clot length of 11.6 ± 6.1 mm in the cases in which no distal embolisation was seen. This may be due to the fact that distal embolisation may, at least in part, be due to the stent-retriever’s length-to-clot-length ratio as has been suggested by Belachew et al. [34]. The fact that the Stream device shortens during expansion may mean that for longer clots, a longer device should be chosen in order to optimise the device/clot interaction, and further studies are required in order to determine if this holds true for the Stream device as it appears to for other devices [35,36,37].

The radial force of a stent-retriever is generally fixed and decreases at increasing diameters. Although techniques for increasing the radial force of standard stent-retrievers have been developed, such as the ‘push and fluff’ technique, these are unlikely to lead to very significant increases in the radial force developed. It has previously been shown that the migration of stent-struts into a clot is determined, at least in part, by the radial force exerted as well as the size of the pores [38]. The radially expandable devices do not share this limitation, and, in fact, the radial force can be increased with manual expansion in a similar manner to, but to a much lesser degree, angioplasty balloons. Conversely, the radial force can also be decreased during the procedure in case excessive resistance is felt. This allows the operator to expand and size the device according to the target vessel; importantly, this can be adapted during the thrombus’s retrieval. This feature may be of particular importance in platelet-rich white thrombi, which prove resistant to standard mechanical thrombectomy approaches. Even in the event of a failure to remove these clots, valuable information can be gleaned from the attempt, as the Stream device is visible along its entire length and, as such, the exact location of the clot can be determined if bailout angioplasty and stenting are to be considered.

In our experience, we have only performed a standard unsheathing and expansion of the Stream device; however, there is some evidence to suggest that repeated inflation and deflation may offer an advantage. In the study conducted by Kara et al. [39], using the Tigertriever, the first-pass recanalisation (mTICI ≥ 2b) rate using a repeated inflation and deflation technique was 47.8% compared to 31.6% when using a standard unsheathing and expansion technique. The authors believe that this might have been due to a combination of factors. Repeated inflation and over inflation may result in the greater enlargement of the cells, allowing more clot fragments to be entrapped within the lumen of the device. The authors also suggest that oversizing causes better apposition of the stent to the vessel wall, which enhances stent–clot interaction. We believe that a further potential reason for this improved recanalisation can be the fact that static expansion will not only potentially compress the clot but also increase the frictional forces between the clot and the vessel wall, whereas repeated inflation and deflation may ‘pull’ the clot away from the vessel wall with each deflation and hence aid in overcoming these frictional forces during retrieval. It remains to be seen whether the repeated expansion technique results in improved recanalisation rates; however, this warrants further investigation with larger cohorts as well as bench-side studies.

Our study has several limitations that are inherent to retrospective observational studies. We did not follow a predefined study protocol that included a fixed number of attempts with the Stream device before switching to a different device/technique, and we did not standardise the other pieces of equipment used. The eTICI scores and follow-up imaging procedures were not adjudicated by an independent core lab.

## 5. Conclusions

The Stream device showed acceptable rates of FPE and mFPE compared to existing devices. Further larger studies are required alongside an understanding of the optimal technique for this device’s use.

## Figures and Tables

**Table 1:** Baseline demographics and medical data.

Baseline Data	*n* = 51
Demographics	
Age	Median 73 (range 51–89)
Female	51% (*n* = 26)
Co-Morbidities	
Smoking	20 (39.2%)
Hypertension	40 (78.4%)
Diabetes Mellitus	13 (25.5%)
Atrial Fibrillation	26 (51%)
Pre-Morbid mRS	
0	51 (100%)

**Table 2:** Baseline clinical and imaging data, including suspected cause of stroke, NIHSS score, clot location, and ASPECT score.

Stroke Data	
NIHSS	Median 17 (range 4–22)
IV tPA	35 (68.6%)
Suspected Cause	
Cardioembolic	27 (52.9%)
Large-artery Atherosclerosis	6 (11.8%)
Mixed	5 (9.8%)
ESUS (Embolic Stroke of Undetermined Source)	13 (25.5%)
Imaging Findings	
Side	
R	29 (56.9%)
L	20 (39.2%)
Mid	2 (3.9%)
Tandem Lesion	
Y	8 (15.7%)
Clot Location	
ICA	16 (31.4%)
M1	29 (56.9%)
M2	4 (7.8%)
BA	2 (3.9%)
Hyperdense Clot	34 (66.7%)
Clot Length	12 ± 6.2 mm (range: 2–26 mm)
ASPECT Score	Median 10 (range: 6–10)

**Table 3:** Procedural data including the model of Stream used, type of anaesthesia, angiographic result following MT, and distal and new-territory embolisation.

Procedural Data	
Anaesthesia	
LA	49 (96.1%)
GA	2 (3.9%)
Distal Aspiration Catheter	51 (100%)
Stream	
Stream	40 (78.4%)
Stream XL	1 (2%)
Stream 17	10 (19.6%)
Angiographic Results	
Median No. of passes	2 (range 1–8)
Median No. of passes with Stream	2 (range 1–4)
FPE (eTICI 2c/3)	13 (25.5%)
Modified FPE (eTICI ≥ 2b)	16 (31.4%)
Final eTICI	
0–2a	5 (9.8%)
2b	3 (5.9%)
2c	4 (7.8%)
3	39 (76.5%)
Distal Embolisation	3 (5.9%)
Embolisation to New territory	0
Bailout Required	*n* = 10
Resistant Clot Device Damage	5 (9.8%) 4 (7.8%)
Intracranial Stent Implanted	1 (1.9%)
Carotid Stent Implanted	2 (3.9%)

**Table 4:** Angiographic and clinical follow-up data.

Follow-up (*n* = 51)	
ASPECT	Median 8 (range 0–10)
sICH	7 (13.7%)
SAH	8 (15.7%)
90-day mRS	
0–2	11 (21.6%)
3–5	18 (35.3%)
6	22 (43.1%)
